# Removal of a Wire Brush Bristle from the Hypopharynx Using Suspension, Microscope, and Fluoroscopy

**DOI:** 10.1155/2015/925873

**Published:** 2015-01-11

**Authors:** Matthew R. Naunheim, Matthew M. Dedmon, Matthew C. Mori, Ahmad R. Sedaghat, Jayme R. Dowdall

**Affiliations:** ^1^Department of Otology and Laryngology, Massachusetts Eye and Ear Infirmary, Boston, MA 02114, USA; ^2^Department of Otolaryngology, Harvard Medical School, Boston, MA 02115, USA; ^3^Division of Otolaryngology, Department of Surgery, Brigham and Women's Hospital, Boston, MA 02115, USA

## Abstract

Wire brush bristles are an increasingly recognized hazard that can present as a foreign body in the aerodigestive tract. Due to their small size and tendency to become embedded in surrounding tissue, these small metallic bristles present a unique operative challenge to otolaryngologists. Here we present a case of a 40-year-old woman who underwent endoscopic extraction of a wire bristle from the posterior pharyngeal wall using suspension, microscopy, and C-arm fluoroscopy. We believe this is the first published case of an endoscopic removal of a buried foreign body in the hypopharynx using these methods of localization concurrently. By leveraging multiple techniques for visualization, surgeons can avoid open exploration while ensuring complete removal of the object. Additionally, this case highlights the importance of regulatory oversight and consumer awareness of the hazards of grill brushes.

## 1. Introduction

**Figure 1:**
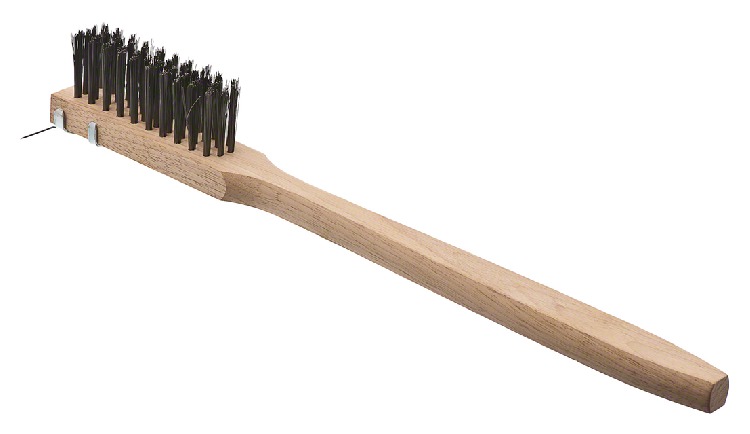
A wire bristle brush used for cleaning grills.

Wire bristle brushes, which are used to clean grills, have small stiff bristles that can break off during use (Figure 1). Reported cases of ingestion of these foreign bodies are relatively rare, but awareness of this risk has increased in recent years [1–4]. Many physicians have called for better warning labels on grill brushes to increase public awareness [2, 3, 5]. Due to these recent reports, the Centers for Disease Control and Prevention (CDC) in 2012 urged physicians and consumers to be aware of wire brush bristle ingestion as a hazard of outdoor grilling and urged that cases be reported to the Consumer Product Safety Commission (CPSC) [6].

Though there is increasing awareness of the dangers of bristles as foreign bodies, removal of these objects from the aerodigestive tract can be exceedingly difficult. These small, thin bristles often cannot be visualized endoscopically and sometimes require removal by open surgical exploration. Here we present a case of wire bristle ingestion and a unique approach to its localization and removal using suspension, microscopy, and intraoperative C-arm fluoroscopy.

## 2. Case Description

**Figure 2:**
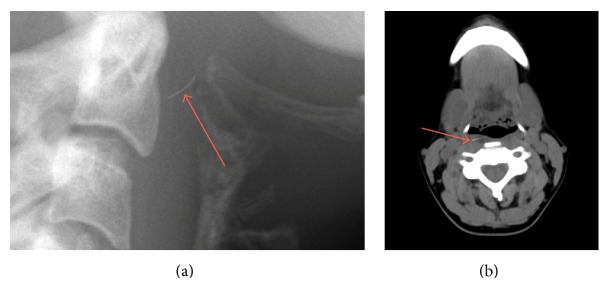
(a) Lateral neck film showing a thin linear density projecting over the base of the dens, with associated prevertebral soft tissue swelling. (b) Nonenhanced axial neck CT with slice thickness of 2 mm demonstrating a 12.3 mm × 1.0 mm linear hyperdense structure in the right posterior pharyngeal wall.

A 40-year-old woman presented to a local emergency room complaining of throat pain. The pain started suddenly while eating chicken breast prepared on a grill, and it worsened with swallowing. She described the pain as constant without radiation. She denied fevers, chills, drooling, intolerance of secretions, and shortness of breath. Her pain persisted for 12 hours, at which point she sought medical attention. A lateral neck X-ray demonstrated a thin linear density projecting over the base of the dens, with associated prevertebral soft tissue swelling (Figure 2(a)). She was transferred to our hospital for further evaluation.

On arrival, the patient continued to note moderate sharp pain in her throat. Physical examination was notable for a normal appearing oral cavity and oropharynx, without trismus, bleeding, or signs of infection. No foreign body was felt on palpation. There was slight tenderness of the right neck overlying the laryngeal cartilage. Flexible fiberoptic exam was notable for right lateral pharyngeal erythema, just above the level of the postcricoid mucosa.

Thin-cut computed tomography demonstrated a 12.3 mm × 1.0 mm linear hyperdense structure in the right posterior pharyngeal wall with mild soft tissue edema (Figure 2(b)). No abscess was seen.

The patient was taken urgently to the operating room for removal of the presumed foreign body.

The patient was intubated with a video laryngoscope to avoid pressure on the posterior pharyngeal wall, which may cause the foreign body to migrate more laterally. A 6.0 endotracheal tube was used. On intubation, a 1 cm area of erythema on the posterior pharyngeal wall was seen, without an obvious point of entry of the foreign body. A tonsil gag was attempted for exposure, but the erythema surrounding the foreign body appeared to be inferior to the level of the base of tongue. Therefore, a Lindholm laryngoscope was used for suspension due to its wide aperture. The microscope was engaged and used to visualize the erythema on the posterior hypopharyngeal wall. The C-arm fluoroscope was placed around the patient's head. The 1 cm area of erythema was palpated with a blunt metallic probe, but this was not in line with the fluoroscopic tip of the object. The blunt tipped probe was therefore moved more superiorly where there was notably less erythema but a small amount of mucosal sloughing. Given the fluoroscopic and microscopic findings, this was determined to be point of penetration. Topical oxymetazoline and submucosal lidocaine with epinephrine were applied. After several minutes, mucosal blanching was seen.

**Figure 3:**
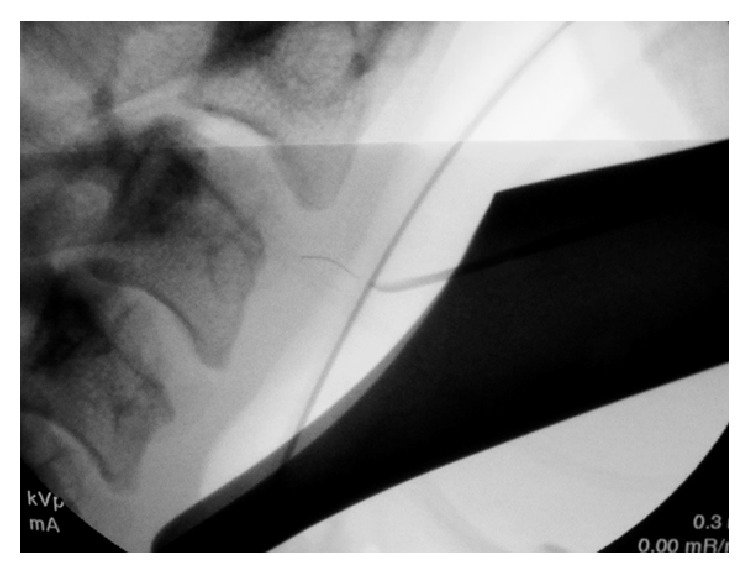
Fluoroscopy verifying position of bristle under tip of blunt probe.

**Figure 4:**
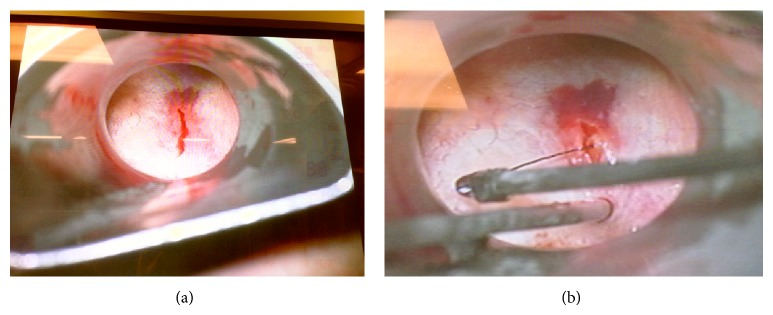
(a) An incision is made in the posterior pharyngeal wall, where the blunt fluoroscopy probe had identified the bristle. (b) Wire being removed from posterior pharyngeal wall.

Using a suction tip, the tip of the foreign body was verified on fluoroscopy to be several millimeters deep to the mucosa (Figure 3). An up-biting scissor was used to make a 4 mm incision in the mucosa of the posterior pharyngeal wall (Figure 4(a)). Pressure was applied medially, and the tip of the foreign object was seen emanating from the right of the incision below the mucosal surface. With downward traction of the medial aspect of the incision, alligator forceps were used to grasp the foreign body and pressure was applied in the medial and superior direction, with simultaneous fluoroscopic and endoscopic visualization (Figure 4(b)). The entire foreign body was removed, verified both grossly and fluoroscopically. The 4 mm incision was left open to heal by secondary intention.

Postoperatively, the patient immediately noted improvement in her pain. She tolerated clear fluids at the same day and was discharged home that evening on a course of antibiotics. She had no complications and has had no further sequelae from her injury.

## 3. Discussion

Foreign bodies of the upper aerodigestive tract are a common complaint seen by otolaryngologists. Sharp objects, like the wire grill brush bristle described in this case, carry significant risk of complications, which include infection, abscess formation, arterial aneurysm, and carotid artery rupture [7–12]. A review of 327 foreign bodies of the upper aerodigestive tract showed an overall 12.6% complication rate in adults, with retropharyngeal abscess being the most common [11]. Fish bones and pharyngeal location were associated with increased incidence of complication.

With the popularity of outdoor grilling, incidence of wire grill brush bristle ingestion has been increasing in recent years [6]. The most common associated presenting symptoms are odynophagia and throat pain [1–4]. A history of grilled meat ingestion and use of a grill brush is particularly important [3]. Bristles can lodge anywhere near the aerodigestive tract and have been reported in various locations, including lingual tonsil [3, 5], base of tongue [2, 3, 12], esophagus [1], vallecula [2], parapharyngeal space [5], and also further down the gastrointestinal tract (small intestine, colon, and omentum) [3]. Some authors suggest that patients be advised to stop ingesting food once bristle ingestion is suspected, as this may force the object further down the digestive tract or deeper into the submucosa [10].

Safe removal of foreign bodies requires precise anatomic localization, whether radiographically or by direct visualization. This is particularly important in cases where the object is embedded deep to the mucosa and when the object is small, both of which our patient demonstrated. Lateral films are often obtained first, though these have limited use for objects that are not radiopaque [13]. If the object cannot be identified on either the physical examination or flexible fiberoptic laryngoscopy, a thin-cut computed tomography (CT) scan should be obtained for both localization and surgical planning. Even on CT, wire brush bristles can be difficult to visualize [3], and many are missed on initial imaging [2, 12].

Apart from traditional imaging, many other techniques for localization of foreign bodies from the aerodigestive tract have been described. These methods include standard laryngoscopy, metal detector [14], flexible bronchoscopy with a channel for forceps [9], microlaryngoscopy [10], and neck exploration [5]. Additionally, C-arm fluoroscopy has been performed for cases of needle breakage in the oral cavity, which are a known complication of inferior alveolar nerve blocks [15].

To our knowledge, this is the first case of concurrent use of suspension, fluoroscopy, and microscopy to locate a foreign body in the upper aerodigestive tract. We think this technique is particularly useful in cases involving wire brush bristles; several case series note that these objects are particularly hard to localize and often require multiple trips to the operating room [2, 5]. This may be due to the bristles' small size and tendency to imbed in surrounding tissue [8].

Fluoroscopy enabled the surgical team to identify the embedded bristle in real time. Using a blunt tipped probe under C-arm, it became apparent that the area of erythema seen on the patient's posterior pharyngeal wall was not the penetration point of the wire bristle. Under the microscope, a previously unnoticed area of sloughing was seen superiorly, and this area was confirmed on fluoroscopy to be the point of entry of the bristle. Thus, suspension combined with fluoroscopy allowed for an image-guided minimally invasive technique performed under direct magnified visualization. We favor this approach to open surgical technique, which otherwise may have been required had the bristle not been located.

In conclusion, wire brush bristles are an increasingly recognized hazard that can present as a foreign body. Otolaryngologists must be aware of this public health issue and should urge careful inspection of grilled foods before ingestion, as well as inspection of grills after they are cleaned with brushes. Removal of these foreign bodies should be done with precise localization and visualization, and fluoroscopy and suspension should be considered key components of the otolaryngologist's armamentarium.
